# Supplementary material for: A mechanistic understanding of collective escape in starling flocks
Source: Commun Biol. 2026 May 18;9:1055. doi: 10.1038/s42003-026-10173-4 (PMC13448496; doi:10.1038/s42003-026-10173-4)
Supplement: Supplementary file 1 [file 42003_2026_10173_MOESM1_ESM.pdf]

nature portfolio

Peer Review File

Towards a mechanistic understanding of collective escape in starlings

Corresponding Author: Dr Marina Papadopoulou

This file contains all reviewer reports in order by version, followed by all author rebuttals in order by version.

Version 0:

Reviewer comments:

Reviewer #1

(Remarks to the Author)
This paper examines collective escape in a model of starling flocking. I find the use of a RoboFalcon to perturb flocks particularly appealing. That said, this approach has been employed previously — including by some of the present authors — and, in fact, the data used here are drawn from their earlier studies.

The authors posit birds can be on one of four “conceptual states,” apparently corresponding to patterns they identify in the video data. This framework enables them to reproduce certain qualitative features observed in the videos. While this is a reasonable strategy, it should be acknowledged that the model appears to recover much of what is already assumed in its construction. For example, the authors interpret the observed “hysteresis” effect as emergent; however, it seems more likely that the predefined state-transition probabilities play a dominant role in producing this effect. Reporting these probabilities would clarify the extent to which the hysteresis is a consequence of the model’s structure rather than an emergent property in the stronger sense.

Although many models can generate realistic-looking collective patterns, this work may indeed offer insight into underlying mechanisms — but the conclusions remain somewhat interpretative and subjective.

Reviewer #2

(Remarks to the Author)
This study provides observations of starling escape behavior enabled by a robotic predator and introduces a novel pattern classification, revealing two distinct patterns can co-occur. Based on the empirical findings, the authors propose a model capable of reproducing the emergence of multiple patterns, including their co-occurrence. The proposed model is intriguing in its incorporation of features such as asynchronous individual reaction times and increased interaction frequency due to state transitions of individuals. I also find the temporal aspect of hysteresis observed in the model particularly interesting. However, I have several concerns, which I outline below.

Major remarks:
1. Regarding the observation:
• The description in lines 88–93 may be somewhat misleading. According to the Methods section, 19 flocks were observed, and co-occurrence was found in 10 of them. However, as currently phrased, the reader may get the impression that co-occurrence was observed in all flocks.
• The sentence "Flocks (N = 10) were stationary..." appears only in the Results section, but is this an important piece of information? If so, it might be better placed in the Methods section. As it stands, it could lead to the misunderstanding that only 10 flocks were analyzed.
• The authors do not explain how the 19 flocks were selected (line 374). Was there a selection criterion? Reference 35 suggests that 54 flocks were filmed—how were the 19 chosen from these?
• The authors mention that co-occurrence was observed in 10 flocks, but do not discuss any properties of these flocks (e.g., their size, number of appearances, etc.). Would it not be useful to include some discussion on this point?

• It would be helpful to clarify what Figure 1C represents. Specifically: Is it derived from time-series data? What is the unit of the timestep? Does it show the duration of co-occurring states observed in the 10 flocks? Related to this, while the authors

mention that two collective patterns co-occurred in at least one step (line 397), the temporal resolution of a "step" is not explained, making it challenging to evaluate how reliably such co-occurrence can be identified.

2. Regarding the model:
• The authors examine the nearest neighbor distance distribution and a neighbor overlap, but would it not be valuable to also consider scale-free correlations (Cavagna et al., 2010)? This could serve as an important measure for validating the model, particularly given that the paper proposes a model of collective response—specifically escape behavior—which is central to its contribution. For example, if differences in range of spatial correlation could be shown between cases where a collective turn occurs alone and cases where it co-occurs with a split, this could provide a more quantitative explanation for instances where information does not propagate rapidly enough through the group. Such an analysis would reinforce the proposed mechanism and substantially enhance the impact of the study.

• Since the model is based on a previous study, the authors provide only a list of parameters. However, it would be helpful to include the actual equations of motion in the supplementary materials.

• It seems that the probabilities of state transitions for individuals are determined by their alertness levels, but a more detailed explanation would make this clearer.
For example, the transition to the Escape state appears to branch into three options (P_ae, P_aa, and P_af) but how exactly are these probabilities determined?

• How is the probability of performing a level-turn vs. a dive in the Escape state defined?
Or is the selection made dynamically depending on whether the individual enters the Escape state when the predator is still beyond the maximum dive distance (in which case a level-turn is executed)?

• Although a topological range is defined for copying, what rule determines whether an individual copies an escape maneuver?
For example, if at least one of the 7 neighbors is performing an escape maneuver, does the individual copy it? Or does it only copy the behavior of its nearest neighbor? If there is a mix of escape types (turn and dive) within the range, how is the maneuver type to copy selected?

3. Title Appropriateness
• The model primarily demonstrates qualitative results (i.e., whether certain patterns emerge), and provides limited quantitative analysis or comparison with existing models. This raises the question of whether the title "revealing mechanisms" fully captures the scope and nature of the findings.

Minor Remarks:
• In the main text, “higher reaction frequency” is denoted as Δt_a, but in the Fig. 5 and Supplementary it appears as Δt_al. Please check the subscripts and ensure consistent notation throughout the manuscript.

Reviewer #3

(Remarks to the Author)
This is a fascinating paper that adds significantly to the current understanding of the collective escape modes of starling flocks, and more generally to the understanding of how perturbations can reveal emergent collective behaviours of animal groups. The key new observation of real starling flocks is that several patterns of collective escape arise simultaneously in different parts of the flock. The authors interpretations of these empirical observations find support in the results of numerical simulations. The results will be of much interest to the many biologists, mathematical biologists and physicists working on collective animal behaviour. The paper is well written and easy to follow. I do, however, have two major comments and two minor comments/suggestions.

1. Major: It should be made clear at lines 54-, 70- and then when introducing StarEscape and at lines 351- that large-scale emergent properties of animal groups (more generally systems) are usually largely independent of the small-scale individual behaviours. Consequently, many different models can result in the same large scale emergent behaviours, and that model agreement with large scale emergent behaviours is no guarantee that the model correctly represents individual behaviours. Claims made at lines 54-, 70- etc. should come with qualifiers, such as ‘Although macroscopic properties do not uniquely constrain the underlying mechanistic interactions, our modelling does suggest…” and “but this accord with observations is no guarantee that the model correctly represents individual behaviours.” Etc.

2. Major: StarEscape The authors need to describe how model predictions for starling flocks in the absence of predators compare with observations, so that readers can gauge whether the model captures correctly the baseline case. For example, is the model in agreement with the 2- and 4-point correlations reported on by Bialek et al. ‘Statistical mechanics for natural flocks of birds’, PNAS 109, 4786-4791 (2012)? Does the model correctly account for the observed scale invariance of long-range correlations? Does the model correctly account for high density borders (Cavagna et al. ‘Diffusion of individual birds in starling flocks’, Proc. Roy. Soc. B. 280, 20122484 (2013)? Etc.

3. Minor: Line 60. Reynolds “Stochasticity may generate coherent motion in bird flocks” Physical Biology 20, 025002 (2023) also modelled the collective escape of starlings, showing how splits can follow flash expansions, and how waves can follow

blackenings. Reynolds also reported In Supplementary Material ala lines 321- and contrary to the claim at lines 324- on model predictions showing a ‘ fast sequence of ‘unstable’ states of the flock during the whole sequence of a pursuit, while the flock transitions from noticing a predator, to escaping, and relaxing after the predator retreats’ . There should also be some mention of Pearce et al. ‘Role of projection in the control of bird flocks’ PNAS 111 10422-10426, 2014 to illustrate the variety of models of murmurations in the literature; and to illustrate comment 1 (above).

4. Minor: At around line 339 some mention could be made of the quite extensive work on how collective mechanical/thermodynamic properties such as tensile strength arise in swarms of midges when they are perturbed (albeit not by predators) in different ways. See papers by Nick Ouellette and co-workers.

Version 1:

Reviewer comments:

Reviewer #2

(Remarks to the Author)
Many thanks for responding so carefully to my comments. The authors have added several new quantitative analyses and results, and the manuscript has been substantially improved.
As a result, I find the manuscript now well suited for publication.
Only a few minor issues remain, as listed below.

Minor:
・I appreciate the authors’ detailed response and the clarifications added to the Methods section regarding the flock selection and the definition of timesteps. These revisions effectively address my concerns about the data processing.
Regarding Fig. 1C, the revised caption is helpful, but it might now contain more detail than necessary for understanding this specific panel. Since Fig. 1C mainly provides an example of the annotated co-occurrence of collective patterns in a single flock, listing the full set of flock IDs in the legend may not be essential. A more concise description such as:
“Example of co-occurrence of collective patterns in the empirical data. Every timestep (2.1–17.1) corresponds to a small time-window (~1 s) in which certain patterns (light coloured blocks) co-occurred in a flock.”
would likely be sufficient. If the authors prefer to retain them for transparency, they might be better placed in the Methods section.

・In Fig. S11, the x-axis scale of panel C appears to differ from the others. Aligning it with the other panels might improve visual consistency.

・Line 158: Pdive = 1 – Pdive. Is this a typo for Pdive = 1 – Pturn?

Reviewer #3

(Remarks to the Author)
The authors have addressed all of my comments comprehensively. I recommend that the paper be accepted for publication.

Open Access This Peer Review File is licensed under a Creative Commons Attribution 4.0 International License, which permits use, sharing, adaptation, distribution and reproduction in any medium or format, as long as you give appropriate credit to the original author(s) and the source, provide a link to the Creative Commons license, and indicate if changes were made.
In cases where reviewers are anonymous, credit should be given to 'Anonymous Referee' and the source.
The images or other third party material in this Peer Review File are included in the article's Creative Commons license, unless indicated otherwise in a credit line to the material. If material is not included in the article's Creative Commons license and your intended use is not permitted by statutory regulation or exceeds the permitted use, you will need to obtain permission directly from the copyright holder.
To view a copy of this license, visit https://creativecommons.org/licenses/by/4.0/

23 February 2026

On behalf of all the authors, I would like to thank the Editor and Reviewers for taking the time to carefully read our work and providing their valuable comments. We have made substantial changes to the manuscript incorporating the feedback provided and dealing with the issues raised by the Reviewers. Please find our answers below in red font. Thank you for the opportunity to submit a revised version of our manuscript for consideration and we look forward to hearing from you regarding our resubmission.

Comments from Associate Editor:

In general, we would ask you to address all the referees' concerns. In particular, you should consider performing the additional quantitative analyses and comparisons with existing models, as mentioned by reviewers #1 and #2.

We revised all sections according to the reviewers' comments. In particular, we introduced 3 new observers (analysis outputs) in our model to extract data on escape probabilities, information transfer, and velocity correlations from a simulation. Based on them we performed additional quantitative analyses and included new subfigures and supplementary figures. As suggested by the Reviewers, we further extended our references to existing models, revised our Introduction and Discussion to emphasise the biological relevance and limitations of models, clarified several points in our Methods and Results, and added a full model description in our Supplementary Material. We further modified our title in response to Reviewer #2. Please find below our answers to each comment of all Reviewers.

Comments from Reviewer #1:

This paper examines collective escape in a model of starling flocking. I find the use of a RoboFalcon to perturb flocks particularly appealing. That said, this approach has been employed previously — including by some of the present authors — and, in fact, the data used here are drawn from their earlier studies.

The authors posit birds can be on one of four "conceptual states," apparently corresponding to patterns they identify in the video data. This framework enables them to reproduce certain qualitative features observed in the videos. While this is a reasonable strategy, it should be acknowledged that the model appears to recover much of what is already assumed in its construction. For example, the authors interpret the observed "hysteresis" effect as emergent; however, it seems more likely that the predefined state-transition probabilities play a dominant role in producing this effect. Reporting these probabilities would clarify the extent to which the hysteresis is a consequence of the model's structure rather than an emergent property in the stronger sense.

We thank the reviewer for their time in reviewing our work. For the case of hysteresis, the predefined probabilities are not actually playing any role, we apologize for any unclarity that supported this conclusion. These state-transition probabilities vary with stress, but reach a parameterized ceiling as

individual stress increases a lot (see Fig. R3 as a reply to Reviewer's #3 comments below). For each simulation we input three transition matrices, one for no, medium, and high stress (Fig. R1). A linear interpolator translate the alertness level of each individual to a probability to transition between states. The probability just controls when a single individual should switch to an escape state, whereas the copying mechanism controls the propagation of this escape information through the flock (see also below our reply to Reviewer's #2 comments on topological range for copying).

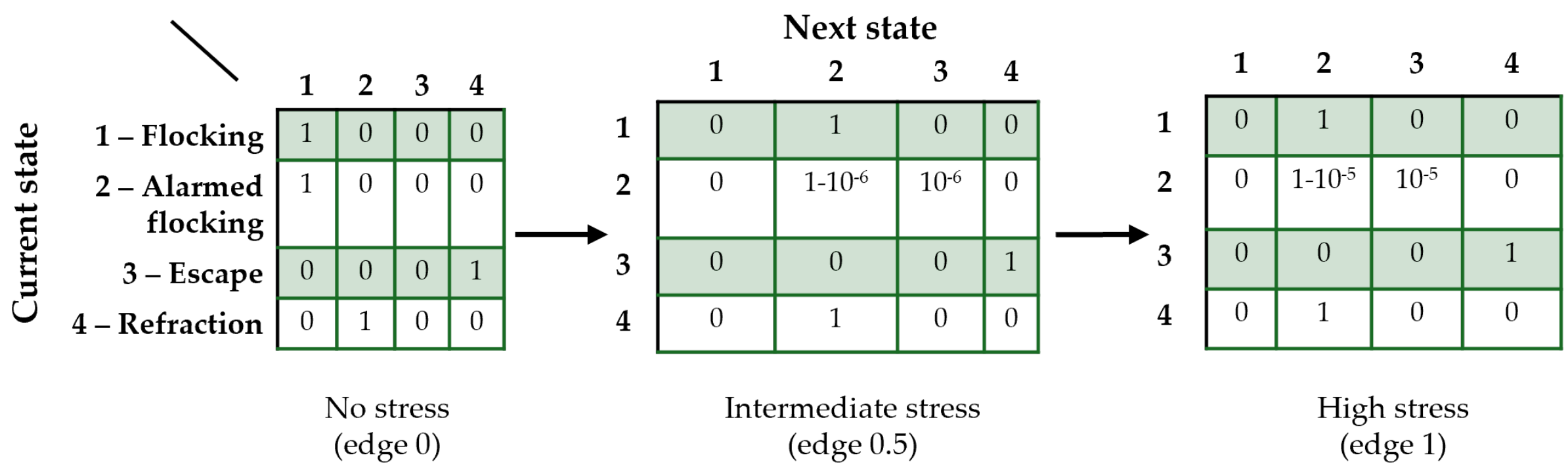

*Figure R1.**Parameterized transition matrices.** A linear interpolator creates a matrix for each agent based on the alertness level of the agent at each update step. The correct probability (based on the current state of the agent) is then used to choose the next state of the agent. Note that states with a set duration (i.e., escape and refraction) use the matrix only after the duration of the state is finished (t_{esc} and t_{ref}).*

To better monitor the interpolated probabilities during a simulation, we further added a new observer in the model that exports the calculated transition probabilities of each agent, allowing for more thorough investigation. In Figure R3 below, we show the timeseries of these probabilities in a simulation, also in relation to distance to the predator and alertness. In our extended model description in the supplementary material (covering also details asked by Reviewer #2), we also provide more details on this technical aspect.

These probabilities thus only affect the duration of the flocking and alarmed states, but do not interfere neither with the reaction frequencies underlying them nor the duration of the escape manoeuvres and refraction period (t_{esc} and t_{ref} parameters, both set to 2 seconds) that are involved in our hysteresis analysis. Lower probabilities in the transition matrices increase the length of the alarmed flocking state prior to a collective escape, while the parameter that controls the decay of alertness (r_{decay}) affects the duration of the alarmed flocking state after the attack. We re-run simulations decreasing these probabilities to allow for longer periods of alarmed flocking and varying group size, updating and revising Figure 4 to improve clarity (with no difference in our findings). We are unsure which other part of the model the Referee sees in what we recover, we agree that this is an important point that we would like to address. Figures R1 and R3 are included as Supplementary Figures S10 and S11 respectively and our text is also revised to improve clarity (lines 267-283).

Although many models can generate realistic-looking collective patterns, this work may indeed offer insight into underlying mechanisms — but the conclusions remain somewhat interpretative and subjective.

We thank the Reviewer for seeing the value in our work and we hope that with this revised version, in which we introduced more quantitative metrics guided by the comments of Reviewers #2 and #3 below, the objectivity of our conclusions is more clear. Specifically, we introduced two extra observers that export

from the model the escape propagation network through which we can measure the speed of information transfer and relate it to the relative position of initiators to the flock centre and predator, which gives us more quantitative proofs to our points (e.g., speed of information transfer and collective turning-split co-occurrence, see also our reply to Reviewer's #2 related comment and new supplementary Figure S6). As we already mention in our Discussion (lines 395-397), we acknowledge the current methodological gap on the automated identification of different patterns. When tackled, it will enable a more complete quantitative and fine scale analysis of details involved in the emergence of different patterns across attacks' specifics, e.g. the exact shape of the flock during an attack from a given direction, but because of the large scale of this complex investigation, tackling all of them is outside the scope of the current manuscript and we hope to address it in the future. We revised our Discussion to emphasize this point (lines 394, 399-400) and we hope that the new metrics and revisions will show that, despite our chosen emphasis on descriptions, the mechanisms we describe are not subjective but set the ground for the next steps, zooming into the complex dynamics of starling flocks.

Comments from Reviewer #2:

This study provides observations of starling escape behavior enabled by a robotic predator and introduces a novel pattern classification, revealing two distinct patterns can co-occur. Based on the empirical findings, the authors propose a model capable of reproducing the emergence of multiple patterns, including their co-occurrence. The proposed model is intriguing in its incorporation of features such as asynchronous individual reaction times and increased interaction frequency due to state transitions of individuals. I also find the temporal aspect of hysteresis observed in the model particularly interesting. However, I have several concerns, which I outline below.

We would like to thank the Reviewer for spending time to carefully examine our work, we highly appreciate the precise feedback and we have revised our manuscript according to all comments, please see our specific answers below.

Major remarks:

1. Regarding the observation:

• The description in lines 88–93 may be somewhat misleading. According to the Methods section, 19 flocks were observed, and co-occurrence was found in 10 of them. However, as currently phrased, the reader may get the impression that co-occurrence was observed in all flocks.

We clarified this in both Results (lines 101: *"We focused our analysis on the flocks in our dataset in which more than one pattern of collective escape co-occurred (N=10)."*) and Methods (lines 439, 445-447): *"From all the derived timeseries of collective events, we kept only the flocks in which two collective patterns co-occurred in at least one step (N = 10)."*).

• The sentence "Flocks (N = 10) were stationary..." appears only in the Results section, but is this an important piece of information? If so, it might be better placed in the Methods section. As it stands, it could lead to the misunderstanding that only 10 flocks were analyzed.

We thank the reviewer for the suggestion, we agree and moved this information to the Methods (lines 425), while revised the Results sentence according to the rest of the comments.

• The authors do not explain how the 19 flocks were selected (line 374). Was there a selection criterion? Reference 35 suggests that 54 flocks were filmed—how were the 19 chosen from these?

We apologize for the confusion, the 19 flocks were all the data from starling flocks. In reference 35, the 54 flocks refer to all species that were included in that work to test the effectiveness of the RobotFalcon as an artificial predator (also corvids, gulls, and lapwings). We added this note in our Methods section (lines 421-422):

'The dataset was collected along with data on other bird species (corvids, gulls and lapwings; by Storms et al. (2022)[39]) between [..]'

• The authors mention that co-occurrence was observed in 10 flocks, but do not discuss any properties of these flocks (e.g., their size, number of appearances, etc.). Would it not be useful to include some discussion on this point?

We thank the reviewer for this suggestion. The flocks in which co-occurrence was observed were in general larger and the RobotFalcon was closer to them and pursuit them for longer, but we don't think we can make a solid claim about this in this current work. As instructed, we added a mention of this in our Discussion (lines 383-386).

• It would be helpful to clarify what Figure 1C represents. Specifically: Is it derived from time-series data? What is the unit of the timestep? Does it show the duration of co-occurring states observed in the 10 flocks? Related to this, while the authors mention that two collective patterns co-occurred in at least one step (line 397), the temporal resolution of a "step" is not explained, making it challenging to evaluate how reliably such co-occurrence can be identified.

We have now clarified how the data underlying the Figure 1C were derived in the Methods (lines 442-447):

'[..] In detail, we factorized each video into a sequence of steps during which a new collective state is observed (a specific pattern appears or disappears, e.g. collective turns last for approximately 1 second). Periods in between patterns of collective escape, during which the flock remained stable in shape, color, and direction of movement, were ignored. From all the derived timeseries of collective events, we kept only the flocks in which two collective patterns co-occurred in at least one step (N = 10). [..]'

and we revised caption 1C and D, hoping that it can also help clarify the evaluation of co-occurrence in the data (lines 167-171):

'[..] Every timestep (2.1 – 17.1) corresponds to a small time-window (~1 seconds) in which a pattern appears or disappears, here focusing on instances that certain patterns (light coloured blocks) co-occurred in a given flock (IDs 2, 4, 6, 8, 11, 12, 13, 14, 16, 17). The data are derived through behavioural sequence analysis on video footage from our ground camera. Timesteps when only a single pattern is observed (e.g., columnar flocking as in B) and time periods without any pattern (forward movement of the flock) are ignored. ***(D)*** *Example of a sequence of different patterns of*

collective escape co-occurring (supplementary Movie S1), in which subgroups (parts of the flock) behaved differently.[..]'

2. Regarding the model:

- The authors examine the nearest neighbor distance distribution and a neighbor overlap, but would it not be valuable to also consider scale-free correlations (Cavagna et al., 2010)? This could serve as an important measure for validating the model, particularly given that the paper proposes a model of collective response—specifically escape behavior—which is central to its contribution. For example, if differences in range of spatial correlation could be shown between cases where a collective turn occurs alone and cases where it co-occurs with a split, this could provide a more quantitative explanation for instances where information does not propagate rapidly enough through the group. Such an analysis would reinforce the proposed mechanism and substantially enhance the impact of the study.

We thank the reviewer for this interesting suggestion. We added a new analysis and observer in our model that exports velocity correlations (a .csv file named 'correlation' within each output folder of a simulation), and validated the presence of scale-free correlations as identified by Cavagna et al. 2010 (Figure R2). We performed more simulations with larger flock sizes and gave each simulated flock a unique ID (this information was added throughout the manuscript).

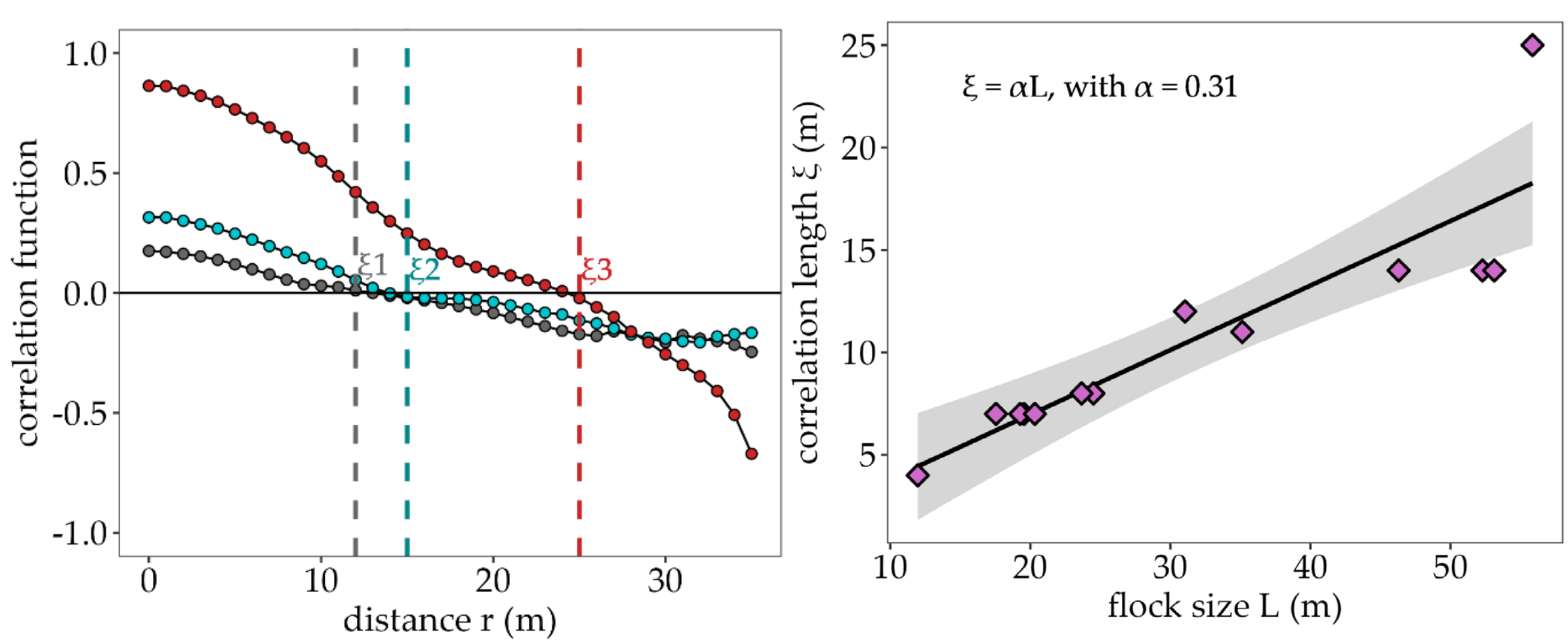

Figure R2. Scale-free correlations in example simulations with 2000 (SF19 and SF20, grey and blue points) and 5000 individuals (SF21, red points), calculated as in [49]. On the left, the vertical lines represent the correlation length ξ of each flock (ξ1, ξ2, ξ3 for flocks SF19, SF20, and SF21, respectively). On the right, each point represents a simulated flock across 7 simulation runs (SF19-25), with the correlation length growing linearly with the size of the flock (Pearson's correlation test: $r = 0.9$, $p < 0.01$). Alpha (α) is close to the value identified in real flocks by Cavagna et al. 2010.

The figures are included as sup-figures Fig.2C-D, and referenced in our revised Results (lines 86-90, 97-102). The observer is mentioned in our new Supplementary text (lines 299-300).

This analysis requires very large group sizes of more than 1000 individuals, something that highly increases the complexity and difficulty of identifying the mechanisms by which different patterns emerge, which is the main focus of our work. We thus did not relate these correlations to different collective patterns, but included another observer that tracks and exports the network of information propagation during an event of collective escape in our simulations (named copychain). Focusing on the cases that collective turns emerge alone and cases that they co-occur with splits, we show the relation between number of splits and speed of information transfer when the group responds with level-turning, also in relation to the parameter for copying a neighbour's escape (topological range for copying). Figure R2 shows results from simulations with 100, 200 and 500 individuals.

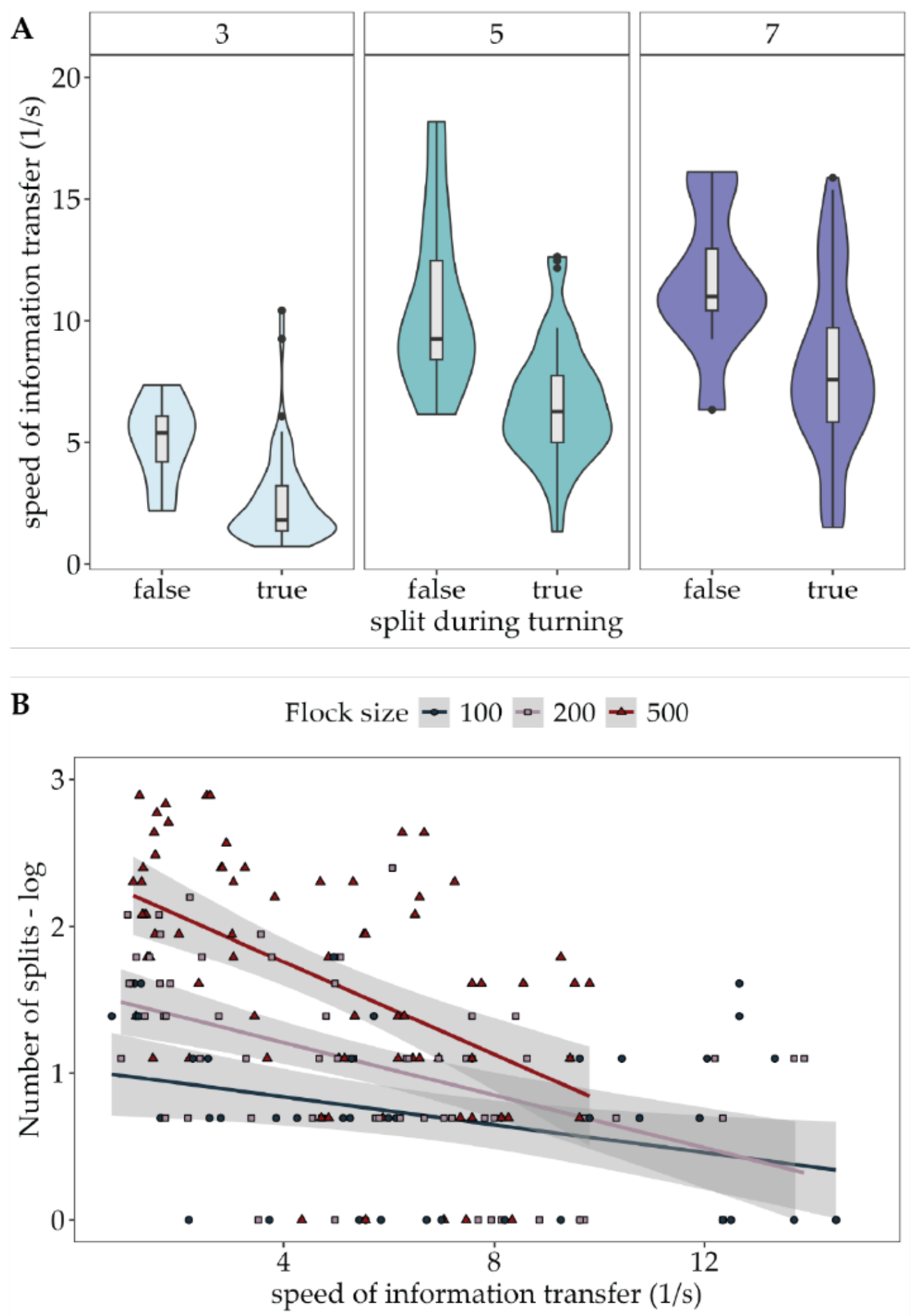

Figure R3. Information transfer and splitting during collective turns. ***A.*** *Slower information transfer (the inverse of the time for an escape maneuver to propagate through the flock) underlies the co-occurrence of splits and collective turns. Each panel corresponds to simulations with different the topological range for copying an escape maneuver (n^{topo}_{copy}; 3, 5 and 7 closest neighbors).* ***B.*** *The faster information propagates through the flock the less splits emerge. Each point represents a single flock during the first attack cycle of a simulation (first time the sturnoids react with a turning escape maneuver away from the predoid). A linear regression fitted to the logged number of splits (across all simulations with varying topological range for copying in which a split occurred; $R^2 = .42$, $p < .001$) shows the significant effect of speed of information transfer ($\beta = 0.96$, $SE = 0.13$, $p < .001$) and flock size ($\beta = 0.002$, $SE = 0.0002$, $p < .001$).*

We included the Figure as supplementary Fig. S6 and refer to it in our Results (lines 219). As the referee mentions, this enables further quantitative analysis based on different question of interest that we think should be part of future work, as noted in our Discussion (lines 399-403):

'The existing monitoring of escape propagation networks, predator-prey details (e.g., relative position of each sturnoid to the predoid), splitting, collective turning, velocity correlations, and flock shape (based on object-oriented box approach) support future endeavors to quantitatively investigate the mechanisms by which flocks transition between collective states and thus quantify self-organized dynamics.'

• Since the model is based on a previous study, the authors provide only a list of parameters. However, it would be helpful to include the actual equations of motion in the supplementary materials.

We extended our Supplementary text to include a full model description using ODD protocol elements and all equations of motion to ensure our model description is standalone (pages 2-9). We further included the main steering force description relating to the agent's motion in the Methods of the main text (lines 577-590), as well as the reference to the full model description (lines 460-461).

• It seems that the probabilities of state transitions for individuals are determined by their alertness levels, but a more detailed explanation would make this clearer. For example, the transition to the Escape state appears to branch into three options (P_ae, P_aa, and P_af) but how exactly are these probabilities determined?

The lack in clarity for the state transitions was also flagged by Reviewer 1. We have now revised our text and included a more detailed explanation of the transitions in the Supplementary Text, including supplementary Figures S10-12 (referred to also in the main text, lines 490, 496, 513, 519). The probabilities are controlled by the alertness (average stress, Fig. R4 and R5A), in combination with the parameters σ_s (parameter for the scaling of stress accumulation), r_{decay} (rate of decay of alertness), and w_{stress} (weighting factor of stress accumulation). P_ae is shown in Fig.R5B. P_aa is effectively 1 - P_ae and P_af is 0 when alertness is high. In low alertness, P_af becomes 1. The transition matrices are initialised as state dependent,

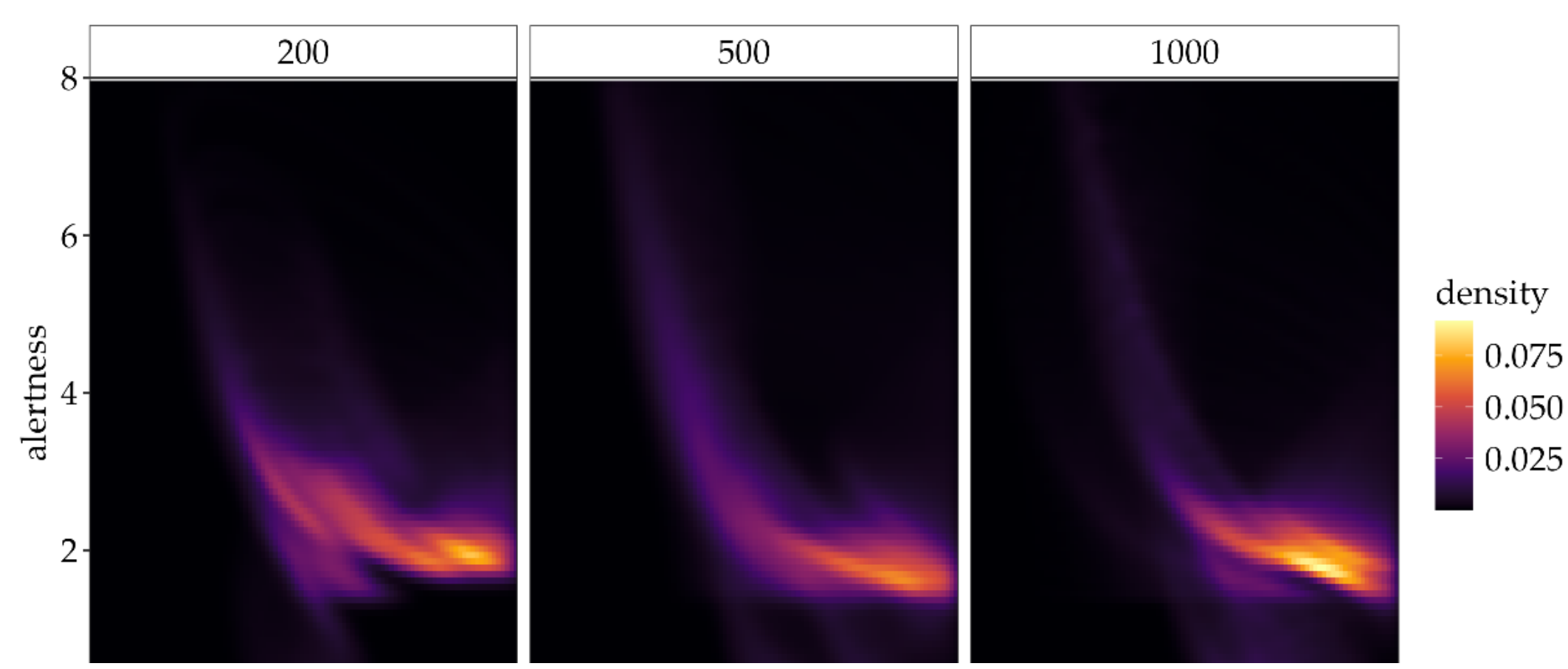

Figure R4. Alertness (or stress) of sturnoids over distance to the predoid during alarmed flocking states in flocks of 200, 500, and 1000 individuals (within 30 meters from the predator).

and a piecewise interpolator is used in the simulation to determine the exact probability from the extremes. See also our answer to Reviewer 1 and Fig. R1.

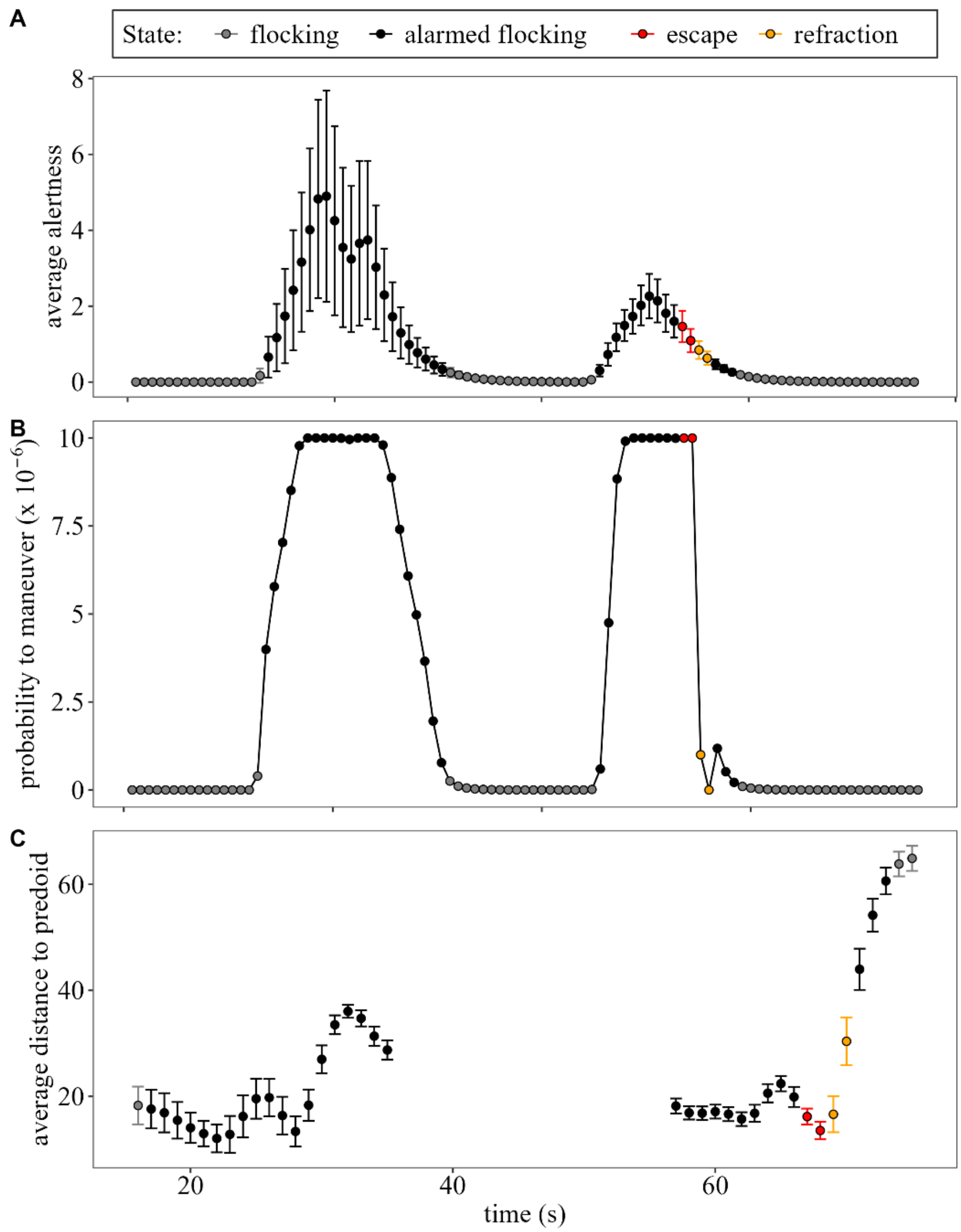

Figure R5. Timeseries in a simulation with 100 individual of average (A) alertness (with error bars indicating the standard deviation across all flock members), (B) probability to perform an escape maneuver (transition from alarmed state to escape) and (C) distance to the predoid (with periods that the predoid is repositioned away from the flock omitted for visualization).

• How is the probability of performing a level-turn vs. a dive in the Escape state defined? Or is the selection made dynamically depending on whether the individual enters the Escape state when the predator is still beyond the maximum dive distance (in which case a level-turn is executed)?

This probability is at the moment a simple parameter, used as 0.5 in the simulations we present in this manuscript. We clarified it in the Results (lines 156-159: *'The selection of which maneuver to perform in the simulations presented here depends on a probability to turn (parameter Pturn) or to dive (Pdive = 1 – Pdive) (see also Supplementary text).*') and in the Methods (lines 613-615: *'A parameterized set of probabilities assigned to each maneuver (Px) controls which evasive action the individual will perform (but see also Further functionality subsection in Supplementary text).'*)

We included the parameter in Supplementary Table S1 (it was mistakenly omitted). However the reviewer is very on point, we do believe that a more dynamic selection would be better, in order to take into account the details of the predator as probably real starlings do. Given that we do not have enough empirical data for this, we included the functionality in the model, i.e. the predefined probabilities can be overwritten when the individual enters the Escape state, but did not use it. This is stated in the *Further functionality* section in the Supplementary text, and we flagged it more clearly in our revised Discussion (lines 390-391):

'Further model functionalities not used in the analysis presented here, such as the deterministic or dynamic selection of escape maneuver, are mentioned in our Supporting Information Text.'

• Although a topological range is defined for copying, what rule determines whether an individual copies an escape maneuver?
For example, if at least one of the 7 neighbors is performing an escape maneuver, does the individual copy it? Or does it only copy the behavior of its nearest neighbor? If there is a mix of escape types (turn and dive) within the range, how is the maneuver type to copy selected?

Yes the copying rule in the model is rather simple, '*if any of the 7 neighbors (within its field of view) is performing an escape maneuver*' the individual copies it. Unfortunately we do not have any more empirical insights to more specifically inform this modelling decision so it remains an assumption. If there is a mix of escape types, based on the software's architecture the individual will copy the manoeuvre of the neighbour that is closer. We revised our Methods to clarify (lines 627-631): *'Note that during copying, an individual copies the type of escape maneuver from any of its interacting neighbors (turn or dive). [..] In the event that an agent has more than 1 neighbors performing a different maneuver, it copies the maneuver of its closest escaping neighbor. '*

3. Title Appropriateness
• The model primarily demonstrates qualitative results (i.e., whether certain patterns emerge), and provides limited quantitative analysis or comparison with existing models. This raises the question of whether the title "revealing mechanisms" fully captures the scope and nature of the findings.

We are open in changing the title of the manuscript, as we agree that it may be perceived as too interpretative. We think that this paper is a step towards a deeper understanding of these very complex mechanisms that we hope to be further investigated in future work (and we hope that this is more apparent by the quantitative analysis included in this revision). We have revised the title to the following:

Towards a mechanistic understanding of collective escape in starlings

We can also have a longer and more specific title if the Editor and Reviewer find it more fitting:

Towards a mechanistic understanding of starling murmurations: self-organized dynamics of collective escape

Minor Remarks:

• In the main text, "higher reaction frequency" is denoted as Δt_a, but in the Fig. 5 and Supplementary it appears as Δt_al. Please check the subscripts and ensure consistent notation throughout the manuscript.

We thank the reviewer for pointing this out, we have corrected it (now Δt_al everywhere).

Comments from Reviewer #3:

This is a fascinating paper that adds significantly to the current understanding of the collective escape modes of starling flocks, and more generally to the understanding of how perturbations can reveal emergent collective behaviours of animal groups. The key new observation of real starling flocks is that several patterns of collective escape arise simultaneously in different parts of the flock. The authors interpretations of these empirical observations find support in the results of numerical simulations. The results will be of much interest to the many biologists, mathematical biologists and physicists working on collective animal behaviour. The paper is well written and easy to follow. I do, however, have two major comments and two minor comments/suggestions.

We thank the author very much for their kind words, we are happy they enjoyed our work. We will answer to the comments below.

1. Major: It should be made clear at lines 54-, 70- and then when introducing StarEscape and at lines 351- that large-scale emergent properties of animal groups (more generally systems) are usually largely independent of the small-scale individual behaviours. Consequently, many different models can result in the same large scale emergent behaviours, and that model agreement with large scale emergent behaviours is no guarantee that the model correctly represents individual behaviours. Claims made at lines 54-, 70- etc. should come with qualifiers, such as 'Although macroscopic properties do not uniquely constrain the underlying mechanistic interactions, our modelling does suggest…" and "but this accord with observations is no guarantee that the model correctly represents individual behaviours." Etc.

We thank the author for the suggestion and agree that this is an important remark that would not be trivial for a non-expert audience. We have thus revised our text in the Introduction (lines 55-61) to clarify:

'[…] although it is important to note that in a simulation, the same macroscopic patterns may emerge from different rules of individual behavior. There is thus no guarantee that models represent correctly individual behavior of animals in nature but they offer a theoretical understanding of what is possible given a set of assumptions. To increase the biological relevance of the theoretical predictions of a model, the local interactions and individual motion of simulated agents can be adjusted to empirical data[21–23]. Model predictions can then in turn drive the collection of more empirical data in order to validate or challenge our theoretical understanding[23].'

2. Major: StarEscape The authors need to describe how model predictions for starling flocks in the absence of predators compare with observations, so that readers can gauge whether the model captures correctly the baseline case. For example, is the model in agreement with the 2- and 4-point correlations reported on by Bialek et al. 'Statistical mechanics for natural flocks of birds', PNAS 109, 4786-4791 (2012)? Does the model correctly account for the observed scale invariance of long-range correlations? Does the model correctly account for high density borders (Cavagna et al. 'Diffusion of individual birds in starling flocks', Proc. Roy. Soc. B. 280, 20122484 (2013)? Etc.

We agree with the reviewer that this is an important point. All the comparisons we present on the collective motion properties of simulated flocks (Figure 2) refer to simulations in the absence of predators, with sturnoids just being in a flocking state. We clarify this now throughout the text (Results lines 186-187, *'the flocks in simulations without a predoid (focusing on the collective motion of the flock) [..]'*, Caption of Fig.2: *'Flocking in StarEscape in the absence of a predator.'*, Methods lines 652-653: *'[..] to validate the collective motion in our model, we run simulations without the predator, and compared [..]'*). Concerning the additional measurements, replying to also Reviewer's 2 suggestion, we investigated the scale-free correlations as shown by Cavagna et al. 2010, and included them in the model and manuscript (see answer above, and new Fig. 2C-D).

Concerning the high density borders, a previous agent-based model of collective motion of starlings (StarDisplay) has captured this property by explicitly making agents sense whether they are at the border or not, and thus increase their attraction to the interior of the flock [2]. To our knowledge, there isn't another individual level rule of flocking that can lead to as dense borders as in the empirical data. We find this relevant and possible to be added to StarEscape as well, but we think that it adds complexity without affecting the mechanisms of the patterns of collective escape we are interested in and focused on in this manuscript (given the topological interactions and information propagation that our model is based on). However, we do make sure that the borders of our simulated flocks are 'sharp' (a characteristic of starling flocks, we revised this detail in the Results lines 190-191) and we avoid 'fuzzy' borders (that are common in agent-based models and can affect information propagation) through our weighted centroid attraction (see also the relevant model description in our new Supplementary Text, lines 60-76).

In addition, we believe that work on the emergence of dense borders is complex enough to be the centre of future work by itself. The definition of a border is not parameter free, i.e. what is counted as 'border' is not a trivial decision and some rather arbitrary assumptions are usually made, especially for non-convex and varying shapes of flocks. We tried to start this investigation in this revision by including a border index in our timeseries observer (see supplementary text *Output* section) and by analysing the convex hull of apparently convex simulated flocks during normal flocking (without escaping the predator). We can thus monitor the nearest neighbour distance of each individual to the closest flock member that is part of the convex hull, seeing a small decrease closer to the border (but note that the flock may not be convex):

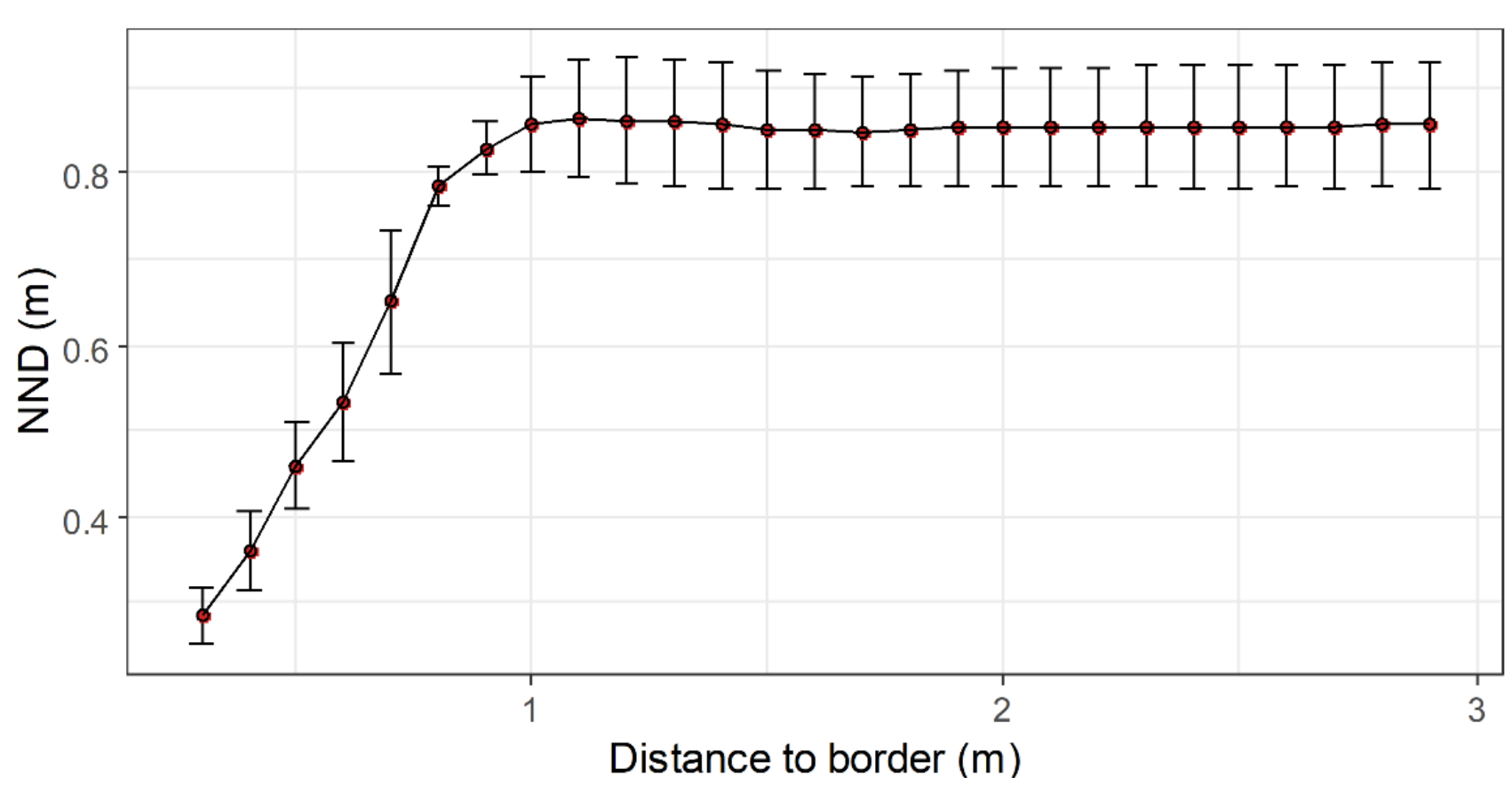

Figure R6.Average nearest neighbor distance of flock members as a function of their distance to a border individual.

Given the above, we decided against including any analysis on this in the main text or adding the extra rule (identified by the previous model) to StarEscape, but highlighted this point and potential for future work in our Discussion (lines 392-394):

'In terms of collective motion, StarEscape can be extended to investigate other characteristic of starling flocks for which underlying mechanisms are not well understood, such as the emergence of high density borders. Concerning future work on collective escape, a [..]'

3. Minor: Line 60. Reynolds "Stochasticity may generate coherent motion in bird flocks" Physical Biology 20, 025002 (2023) also modelled the collective escape of starlings, showing how splits can follow flash expansions, and how waves can follow blackenings. Reynolds also reported In Supplementary Material ala lines 321- and contrary to the claim at lines 324- on model predictions showing a ' fast sequence of 'unstable' states of the flock during the whole sequence of a pursuit, while the flock transitions from noticing a predator, to escaping, and relaxing after the predator retreats' . There should also be some mention of Pearce et al. 'Role of projection in the control of bird flocks' PNAS 111 10422-10426, 2014 to illustrate the variety of models of murmurations in the literature; and to illustrate comment 1 (above).

We thank the reviewer for binging to our attention these references. We have revised the Introduction (lines73-75) and the Discussion (lines 336-337), revising our terminology to avoid confusion and including the mentioned works (references 27 and 34 in revised the manuscript).

4. Minor: At around line 339 some mention could be made of the quite extensive work on how collective mechanical/thermodynamic properties such as tensile strength arise in swarms of midges when they are perturbed (albeit not by predators) in different ways. See papers by Nick Ouellette and co-workers.

We thank the reviewer for the suggestion, we incorporated this interesting side of collective response to perturbations in our Discussion (lines 359).

29 March 2026

On behalf of all the authors, I would like to thank the Editor and Reviewers for taking the time to carefully examine the revised version of our manuscript and for their positive response. We have revised the manuscript according to the minor remarks of Reviewer #2 (the caption of Fig. 1C, supplementary Fig. S11, and typo), and adjusted it to the journal's final revision instructions, including shortening the abstract to 150 words and updating our Code and Data Availability sections.

Thank you for your interest and your contribution into improving our work and we look forward to hearing from you regarding our resubmission.

Kind regards,
Marina Papadopoulou
