# Supplementary material for: A mechanistic understanding of collective escape in starling flocks
Source: Commun Biol. 2026 May 18;9:1055. doi: 10.1038/s42003-026-10173-4 (PMC13448496; doi:10.1038/s42003-026-10173-4)
Supplement: Supplementary file 3 [file 42003_2026_10173_MOESM3_ESM.pdf]

Corresponding author(s): Marina Papadopoulou

Last updated by author(s): Mar 29, 2026

Reporting Summary

Nature Portfolio wishes to improve the reproducibility of the work that we publish. This form provides structure for consistency and transparency in reporting. For further information on Nature Portfolio policies, see our Editorial Policies and the Editorial Policy Checklist.

Statistics

For all statistical analyses, confirm that the following items are present in the figure legend, table legend, main text, or Methods section.

n/a	Confirmed	
☐	☒	The exact sample size (*n*) for each experimental group/condition, given as a discrete number and unit of measurement
☐	☒	A statement on whether measurements were taken from distinct samples or whether the same sample was measured repeatedly
☒	☐	The statistical test(s) used AND whether they are one- or two-sided<br>*Only common tests should be described solely by name; describe more complex techniques in the Methods section.*
☒	☐	A description of all covariates tested
☒	☐	A description of any assumptions or corrections, such as tests of normality and adjustment for multiple comparisons
☐	☒	A full description of the statistical parameters including central tendency (e.g. means) or other basic estimates (e.g. regression coefficient) AND variation (e.g. standard deviation) or associated estimates of uncertainty (e.g. confidence intervals)
☒	☐	For null hypothesis testing, the test statistic (e.g. *F*, *t*, *r*) with confidence intervals, effect sizes, degrees of freedom and *P* value noted<br>*Give P values as exact values whenever suitable.*
☒	☐	For Bayesian analysis, information on the choice of priors and Markov chain Monte Carlo settings
☒	☐	For hierarchical and complex designs, identification of the appropriate level for tests and full reporting of outcomes
☒	☐	Estimates of effect sizes (e.g. Cohen's *d*, Pearson's *r*), indicating how they were calculated

Our web collection on statistics for biologists contains articles on many of the points above.

Software and code

Policy information about availability of computer code

Data collection	Simulated data were extracted by our agent-based model StarEscape (custom code in C++), that it's 1st version (v.1.0) is presented and published with this manuscript. No software was used for the collection of the empirical data.
Data analysis	The software Adobe Premier Pro was used to mark and analyse the empirical data (video footage). Custom code in R (v. 4.3.2) was used for the analysis of both empirical and simulted data, published with this manuscript.

For manuscripts utilizing custom algorithms or software that are central to the research but not yet described in published literature, software must be made available to editors and reviewers. We strongly encourage code deposition in a community repository (e.g. GitHub). See the Nature Portfolio guidelines for submitting code & software for further information.

Data

Policy information about availability of data

All manuscripts must include a data availability statement. This statement should provide the following information, where applicable:

- Accession codes, unique identifiers, or web links for publicly available datasets
- A description of any restrictions on data availability
- For clinical datasets or third party data, please ensure that the statement adheres to our policy

All simulated and extracted empirical data will be available on a Zenodo repository upon acceptance.

Research involving human participants, their data, or biological material

Policy information about studies with human participants or human data. See also policy information about sex, gender (identity/presentation), and sexual orientation and race, ethnicity and racism.

Reporting on sex and gender	NA
Reporting on race, ethnicity, or other socially relevant groupings	NA
Population characteristics	NA
Recruitment	NA
Ethics oversight	NA

Note that full information on the approval of the study protocol must also be provided in the manuscript.

Field-specific reporting

Please select the one below that is the best fit for your research. If you are not sure, read the appropriate sections before making your selection.

☐ Life sciences ☐ Behavioural & social sciences ☒ Ecological, evolutionary & environmental sciences

For a reference copy of the document with all sections, see nature.com/documents/nr-reporting-summary-flat.pdf

Ecological, evolutionary & environmental sciences study design

All studies must disclose on these points even when the disclosure is negative.

Study description	Video footage of starling flocks attacked by a robotic falcon in the field were collected through a ground camera and a camera on top of the robot.
Research sample	Videos of flocks of wild European starlings (Sturnus vulgaris) during a pursuit by the robotic falcon.
Sampling strategy	All flocks that were encountered during the fieldwork were included (but see Disturbance section below for exceptions).
Data collection	The video footage was collected by Rolf Storms and field student assistants through a ground camera (Sony FDR-AX53 4K Camcorder, 50 fps). The data collection team included the pilot of the robotic falcon (Robert Musters), a camera operator and a coordinator that was guiding the pilot and collecting audio recordings of the pursuit. The camera on top of the robotic falcon was a Runcam micro swift2 (30 fps). The flocks were first spotted on the ground and then a detterence experiment was initiated. The robot was steered to pursuit the flock (keeping a close distance behind it) and occasionally perfoming attacks (diving in the flock), similar to the hunting of a real falcon. The video recordings were stopped when a flock was far away from the site and couldn't be seen (> 1 km).
Timing and spatial scale	Footage was collected between the 25th of February 2019 and 22nd of November 2019, excluding the breeding season of the birds (April to July), with an average of 6 days of fieldwork per month.
Data exclusions	Footage in which the flock quickly flew away from the study site without the robot getting close were not included in our analysis.
Reproducibility	The falcon was successful in eliciting escape responses from the flocks (see also Storms R.F. et al. 2022 Journal of the Royal Society Interface).
Randomization	The flocks that were attacked were randomly encountered sitting on the ground within the study area (private agricultural fields) and were chased away after. We cannot know whether individuals from these flocks have been included in the data from a previous instance.
Blinding	The footage was analyzed independently by two team-members and the identification of patterns of collective escape in it was validated.

Did the study involve field work? ☒ Yes ☐ No

Field work, collection and transport

Field conditions	We performed experiments only in the absence of rain and with winds lower than 6 on the Beaufort scale.

Location	The data were collected in an agricultural area close to Workum in the north of the Netherlands (52°59' N–5°27' E).
Access & import/export	Our samples were video footage. Permission to deter the birds and collect video footage was provided by Peet Sterkenburgh.
Disturbance	The starling flocks that were found sitting on the ground were deterred by the robotic falcon, without the robot getting dangerously close to any individual. If other bird species that shouldn't be disturbed were present, the data collection was not performed.

Reporting for specific materials, systems and methods

We require information from authors about some types of materials, experimental systems and methods used in many studies. Here, indicate whether each material, system or method listed is relevant to your study. If you are not sure if a list item applies to your research, read the appropriate section before selecting a response.

Materials & experimental systems

n/a	Involved in the study
☒	☐ Antibodies
☒	☐ Eukaryotic cell lines
☒	☐ Palaeontology and archaeology
☐	☒ Animals and other organisms
☒	☐ Clinical data
☒	☐ Dual use research of concern
☒	☐ Plants

Methods

n/a	Involved in the study
☒	☐ ChIP-seq
☒	☐ Flow cytometry
☒	☐ MRI-based neuroimaging

Animals and other research organisms

Policy information about studies involving animals; ARRIVE guidelines recommended for reporting animal research, and Sex and Gender in Research

Laboratory animals	The study did not involve laboratory animals.
Wild animals	Flocks of European starlings in the north of the Netherlands were approached and recorded using a robotic falcon and a ground camera. The flocks became airborne and were pursued by the robot until leaving the site. No animals were caught or harmed.
Reporting on sex	We worked with wild bird populations so such information was not collected.
Field-collected samples	The study did not involve samples collected in the field.
Ethics oversight	The fieldwork was carried out in agreement with the rules of the University of Groningen Animal Welfare Committee. The target species is common in the area with frequent interactions with humans. Peet Sterkenburgh provided us with the permissions to interact with the specific bird species within specific areas in Workum.

Note that full information on the approval of the study protocol must also be provided in the manuscript.

Plants

Seed stocks	*Report on the source of all seed stocks or other plant material used. If applicable, state the seed stock centre and catalogue number. If plant specimens were collected from the field, describe the collection location, date and sampling procedures.*
Novel plant genotypes	*Describe the methods by which all novel plant genotypes were produced. This includes those generated by transgenic approaches, gene editing, chemical/radiation-based mutagenesis and hybridization. For transgenic lines, describe the transformation method, the number of independent lines analyzed and the generation upon which experiments were performed. For gene-edited lines, describe the editor used, the endogenous sequence targeted for editing, the targeting guide RNA sequence (if applicable) and how the editor was applied.*
Authentication	*Describe any authentication procedures for each seed stock used or novel genotype generated. Describe any experiments used to assess the effect of a mutation and, where applicable, how potential secondary effects (e.g. second site T-DNA insertions, mosiacism, off-target gene editing) were examined.*
